# Clinical Significance of Ischemia-Modified Albumin in the Diagnosis of Doxorubicin-Induced Myocardial Injury in Breast Cancer Patients 

**DOI:** 10.1371/journal.pone.0079426

**Published:** 2013-11-04

**Authors:** Yinghuan Ma, Wanjun Kang, Yongxin Bao, Fubin Jiao, Yiran Ma

**Affiliations:** 1 Department of Cancer, The 463rd Hospital of the Chinese PLA, Shenyang, Liaoning, People’s Republic of China; 2 Division of Health, Bureau of Guard, General Advisor Office of Chinese PLA, Beijing, People’s Republic of China; 3 Department of Transfusion, The First Hospital of China Medical University, Shenyang, Liaoning, People’s Republic of China; Virginia Commonwealth University, United States of America

## Abstract

**Background:**

Ischemia-modified albumin is an altered serum albumin that forms under conditions of oxidative stress, a state also associated with doxorubicin-induced myocardial injury.

**Objective:**

The aim of this study was to better assess diagnostic and prognostic significance of ischemia-modified albumin in patients with breast cancer undergoing doxorubicin chemotherapy.

**Methods:**

Blood samples were collected from 152 breast cancer patients before and after each cycle of doxorubicin chemotherapy to measure the serum levels of ischemia-modified albumin, cardiac troponin T and creatine kinase-MB. We also monitored cardiac function during a 12 month follow-up.

**Results:**

There was a significant difference in ischemia-modified albumin levels before and after each cycle of chemotherapy and the ischemia-modified albumin concentration positively correlated with the cumulative dose of doxorubicin (*r* = 0.212, *P* < 0.05). The combination of ischemia-modified albumin with cardiac troponin T and creatine kinase-MB increased the sensitivity to 0.920 and the specificity to 0.830 in the diagnosis of doxorubicin-induced myocardial injury. The optimal cutoff for ischemia-modified albumin concentration was 112.09 U/ml. The rate of change for ischemia-modified albumin levels correlated negatively with the rate of change for left ventricular ejection fraction at one year (*r* = –0.221, *P* < 0.05).

**Conclusion:**

Ischemia-modified albumin may be a clinically potential new marker for diagnosing doxorubicin-induced myocardial injury, and is helpful to predict long-term impairment of cardiac function.

## 1. Introduction

Doxorubicin (DOX) is an anthracycline antibiotic with strong anticancer activity first extracted from *Streptomyces peucetius* *var.* *caesius* in 1969 [1,2]. Doxorubicin exerts its cytotoxic effects on tumors by entering cells and combining with chromosomes. The planar ring of Doxorubicin intercalates with a DNA base pair and forms a complex that impairs the synthesis of DNA, RNA and protein by inducing topoisomerase II to cleave DNA and damage its tertiary structure.

Secondary myocardial injury was identified shortly after widespread clinical use of DOX as a chemotherapy drug [3]. The symptoms and signs of DOX -induced myocardial injury are similar to dilated cardiomyopathy: chest pain, dyspnea, tachycardia or bradycardia, pericardial effusion and heart failure. Once congestive heart failure develops, the mortality rate is 48% [4,5] and patients with DOX-induced myocardial injury represent a diagnostic challenge. Currently, the diagnosis of DOX-induced myocardial injury is based on the symptoms, electrocardiogram (ECG) and cardiac troponin-T (cTnT) and creatine kinase-MB (CK-MB) fraction levels. However, clinical symptoms may be atypical and unreliable, ECG changes are transient and biochemical tests lack sufficient sensitivity and accuracy. A better method is needed to aid in the diagnosis of DOX-induced myocardial injury.

Ischemia-modified albumin (IMA) can be detected in the early stages of myocardial ischemia before the onset of myocardial necrosis, and is the first serum marker for myocardial ischemia to be approved by the United States Food and Drug Administration [6,7]. Serum IMA elevations occur early in the development of myocardial ischemia, and IMA is a sensitive indicator of injury with high positive and negative predictive values [6,7]. Droy and colleagues [8] were the first to show that IMA formation is directly linked with the presence of reactive oxygen species (ROS), especially the hydroxyl radical (•OH). IMA is formed when serum albumin passes through ischemic tissues where the oxidative stress induces an increase in oxygen free radicals, which results in IMA formation *in vivo* by oxidative modification of serum albumin [9-11]. The precise mechanisms underlying DOX-induced myocardial injury are believed to involve free radicals, mitochondrial damage and calcium overload, with oxidative stress caused by free radicals as the most widely accepted [12,13]. However, serum IMA levels in patients undergoing DOX chemotherapy have yet not to be investigated.

The aim of this study was to investigate serum IMA levels in patients with breast cancer undergoing DOX chemotherapy and to evaluate the potential value of IMA as a diagnostic indicator. We measured serum IMA, cTnT and CK-MB in patients with breast cancer before and after each cycle of DOX chemotherapy and recorded subsequent changes in their long-term cardiac function. Furthermore, we assessed the performance of IMA levels by receiver operating characteristic curves (ROC) to determine whether this indicator might be helpful in the diagnosis of DOX -induced myocardial injury.

## 2. Materials and Methods

### 2.1: Patients

In the 463rd Hospital of the Chinese PLA between March 2007 and September 2011, we recruited 152 patients with stage I and II breast cancer scheduled for surgery and receiving the FAC chemotherapy protocol (0.8 g/m^2^ cyclophosphamide + 50 mg/m^2^ DOX + 1.0 g/m^2^ 5-fluorouracil, one cycle). Each patient received 1–6 cycles of chemotherapy and the median age was 56 (range, 35–68) years. After each cycle, an ECG was performed, and CK-MB, cTnT and IMA levels were measured. If a patient was unable to tolerate chemotherapy or myocardial injury occurred, this cycle of chemotherapy was stopped and alternative treatments were sought, but the serum samples of every chemotherapy cycle before this cycle of chemotherapy was collected and stored. Written informed consent was obtained from all patients, and the study was approved by the 463rd Hospital of the Chinese PLA Ethics Committee. 

We used the following exclusion criteria: an abnormal ECG, cTnT or CK-MB at initial screening; pregnancy; active infection; musculoskeletal disease; hematological disease; immune disease; liver or kidney disease; serum albumin concentration outside the range of (35.0~55.0g/L); a history of trauma or surgery in the two weeks prior to the initial assessment.

### 2.2: Diagnosis of DOX-induced myocardial injury

A patient was considered to have DOX-induced myocardial injury if they developed: symptoms of chest pain or dyspnea; an abnormal ECG; increased serum CK-MB or cTnT; or a decrease in LVEF ≥ 10% compared with baseline during or following chemotherapy [14].

Abnormal ECG definition: The changes of ST-T, T and Q-T in ECG occurred for patients before and after chemotherapy, and the changes can’t be restored immediately.

### 2.3: Allocation of clinical groups

#### Pre-chemotherapy group and post-chemotherapy group

The serum samples of patients receiving the FAC chemotherapy protocol were divided into pre-chemotherapy group and post-chemotherapy group. The 152 serum samples of 152 patients before chemotherapy belong to pre-chemotherapy group, and serum samples of patients after chemotherapy were divided into 6 groups according to the cycle of chemotherapy: 

The first cycle of chemotherapy group , the second cycle of chemotherapy group , the third cycle of chemotherapy group , the fourth cycle of chemotherapy group , the fifth cycle of chemotherapy group , and the sixth cycle of chemotherapy group. 

#### Myocardial injury group and no myocardial injury group

The serum samples of patients after each cycle of chemotherapy were divided into myocardial injury group and no myocardial injury group according to the diagnosis criteria of myocardial injury previously noted (see section 2.2 in method).

#### Group A (mean IMA > the optimal cutoff) and Group B (mean IMA < the optimal cutoff)

The 59 patients from no myocardial injury group undergoing all 6 chemotherapy cycles were followed-up for 1 year, who received no other drugs with the potential to cause myocardial injury. The ROC curve of the relationship between the serum IMA concentrations of post-chemotherapy and DOX-induced myocardial injury was plotted and an IMA cutoff value was calculated. We divided the serums of 59 patients who were followed up for one year into group A (mean IMA concentration / patient > the optimal cutoff) and Group B (mean IMA concentration /patient < the optimal cutoff) by comparing the mean IMA concentration of post-chemotherapy with the optimal cutoff.

Left ventricular ejection fraction (LVEF) was measured in the 59 patients.

### 2.4: Sample Collection and Measurement

An 8 ml sample of peripheral venous blood was collected from patients at 4 hours after each cycle of chemotherapy, and stored in a yellow-topped tube with the inert separation gel and coagulant. After centrifugation, the serums were decanted and measured immediately or stored at -80 °C. Serum IMA level was measured by albumin cobalt test kits according to the manufacturer’s instructions and using their reagents and equipment (Changsha Yikang Science Technique Development Co. Ltd., China). Serum CK-MB and cTnT level was measured by electrochemiluminescence kits according to the manufacturer’s instructions and using their reagents and equipment (Roche, Germany). All assays were performed in duplicate. The normal reference value for CK-MB was < 25 U/L and the normal reference value for cTnT was < 0.05 ng/ml, with ≥ 0.1 ng/ml considered positive. LVEF was measured by ECG using a 4VI-C probe, with a frequency of 1.7–3.4 MHz, and frame frequency of 40–90 frame/s (Acuson Sequoia, Siemens, Germany).

### 2.5: Statistical analysis

All data were analyzed using SPSS 13.0 software (SPSS, Chicago, USA). The distribution of the IMA values was tested for normality using the Kolmogorov-Smirnov test. Normally distributed data were expressed as mean ± standard deviation (SD), and compared using the t-test. Data that were not normally distributed were presented as median (2.5th–97.5th percentile), and compared using the Wilcoxon rank sum test. The correlation of the cumulative dose of DOX and serum IMA measured at each cycle of chemotherapy was tested by the linear correlation analysis. The sensitivity and specificity of IMA, the areas under the curve (AUC) and the optimal cutoff concentration value are calculated by ROC curves in SPSS 13.0 software [15]. IMA, cTnT, CK-MB, and IMA + cTnT + CK-MB in the detection of DOX-induced myocardial injury were analyzed by ROC, 136 patients with no myocardial injury as a negative group and 16 patients with myocardial injury as a positive group. A ROC curve was drawn to subdivide the groups according to the IMA concentration and to allow analysis of the relationship between IMA concentration and DOX-induced myocardial injury. A *P* value of < 0.05 was considered statistically significant. Spearman univariate correlation analysis was conducted between the rate of change for IMA with the rate of change for LVEF. 

## 3. Results

**Figure 1:**
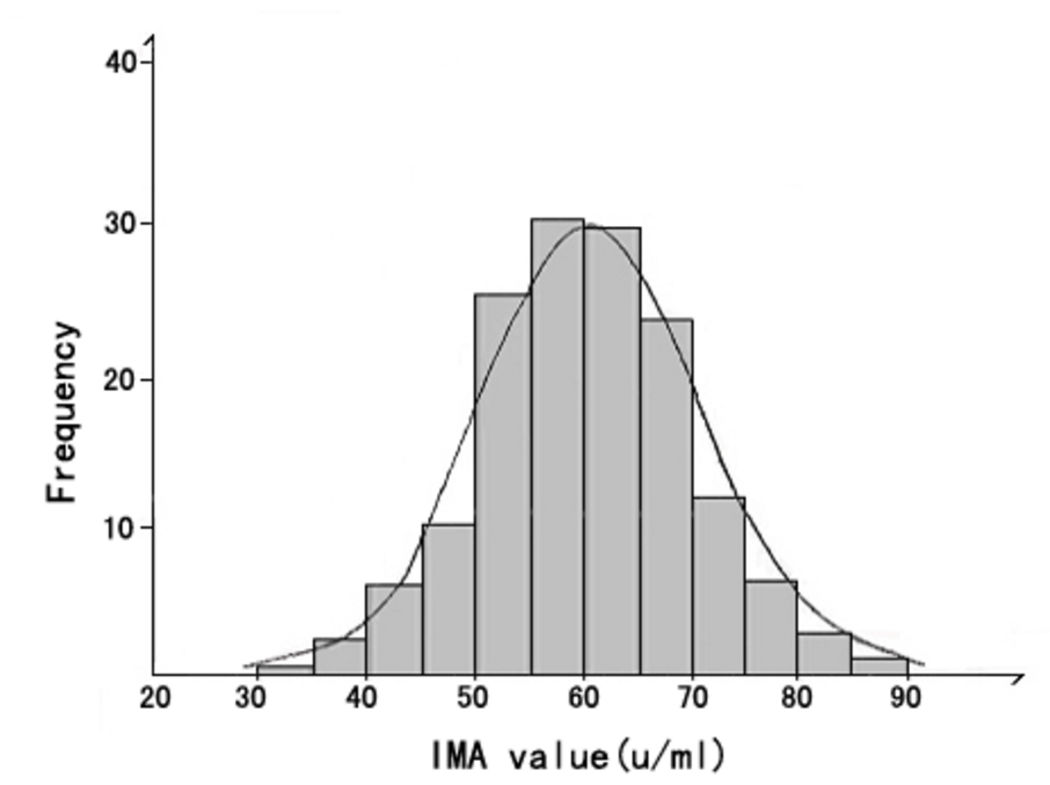
The distribution of serum IMA concentration data from pre-chemotherapy. The distribution of serum IMA concentration data from pre-chemotherapy is normal. The mean IMA concentration was (59.2 ± SD 10.9) U/ml.

We found a normal distribution for the pre-chemotherapy serum IMA concentration data using the Kolmogorov-Smirnov test. The mean IMA concentration was (59.2 ± SD 10.9) U/ml (Figure 1). 152 patients received the first cycle of chemotherapy with the DOX dose as 50mg/m^2^, and 2 patients happened myocardial injury. The serum mean IMA concentration (63.2±13.2)U/ml of 152 patients after the first cycle of chemotherapy and the serum mean IMA concentration (59.2 ± SD 10.9) U/ml of 152 patients before chemotherapy were analyzed by paired t-test, and there are significant difference (*t*_*1*_=2.88, *p*_*1*_=0.002). 147 patients received the second cycle of chemotherapy with the DOX dose as 100mg/m^2^, and only one patient happened myocardial injury. The serum mean IMA concentration (73.8±14.3)U/ml of 147 patients after the second cycle of chemotherapy and the serum mean IMA concentration (57.8 ± SD 10.5) U/ml of these 147 patients before chemotherapy were analyzed by paired t-test, and there are significant difference (*t*_*2*_=8.63, *p*_*2*_=0.000). In the third cycle of chemotherapy, 109 patients received DOX chemotherapy with the dose as 150mg/m^2^, and myocardial injury occurred in 4 patients. The serum mean IMA concentration (82.5±17.6)U/ml of 109 patients after the third cycle of chemotherapy and the serum mean IMA concentration (60.5 ± SD 11.3) U/ml of 109 patients before chemotherapy were analyzed by paired t-test, and there are significant difference (*t*_*3*_=13.26, *p*_*3*_=0.000). 97 patients received the fourth cycle of chemotherapy with the DOX dose as 200mg/m^2^, and 3 patients happened myocardial injury. The serum mean IMA concentration (85.3±18.6)U/ml of 97 patients after the fourth cycle of chemotherapy and the serum mean IMA concentration (57.6 ± SD 11.8) U/ml of 97 patients before chemotherapy were analyzed by paired t-test, and there are significant difference (*t*_*4*_=16.61, *p*_*4*_=0.000). 80 patients received the fifth cycle of chemotherapy with the DOX dose as 250mg/m^2^, and 2 patients appeared myocardial injury. The serum mean IMA concentration (88.2±20.2)U/ml of 80 patients after the fifth cycle of chemotherapy and the serum mean IMA concentration (58.4 ± SD 9.8) U/ml of 80 patients before chemotherapy were analyzed by paired t-test, and there are significant difference (*t*_*5*_=16.14, *p*_*5*_=0.000). 73 patients received the sixth cycle of chemotherapy with the DOX dose as 300mg/m^2^, and 4 patients appeared myocardial injury. The serum mean IMA concentration (93.6±19.7)U/ml of 73 patients after the sixth cycle of chemotherapy and the serum mean IMA concentration (57.9 ± SD 12.1) U/ml of 73 patients before chemotherapy were analyzed by paired t-test, and there are significant difference (*t*_*6*_=17.18, *p*_*6*_=0.000)_○_

The serum samples of patients after each cycle of chemotherapy were divided into myocardial injury group and no myocardial injury group according to the diagnosis criteria of myocardial injury previously noted (see section 2.2 in method).The difference of IMA concentration in serum samples between myocardial injury group and no myocardial injury group in each cycle of chemotherapy was analyzed by t-test, there was significant difference for all five cycles of chemotherapy. The data from the second cycle of chemotherapy were excluded because only one patient developed into myocardial injury which is not suitable for t-test (Table 1).

**Table 1:** IMA concentration in serum samples in myocardial injury group and no myocardial injury group for each cycle of chemotherapy.

Groups		n	The mean IMA concentration(U/ml)	SD	*t* value	P value
The first cycle of chemotherapy group	myocardial injury group	2	108.3	5.3	*t*_*1*_=5.035	P_*1*_=0.000
	no myocardial injury group	150	62.2	12.9		
The third cycle of chemotherapy group	myocardial injury group	4	128.4	10.3	*t*_*2*_=9.125	P_*2*_=0.000
	no myocardial injury group	105	70.1	12.6		
The fourth cycle of chemotherapy group	myocardial injury group	3	133.6	20.1	*t*_*3*_=5.742	P_*3*_=0.000
	no myocardial injury group	94	80.7	15.6		
The fifth cycle of chemotherapy group	myocardial injury group	2	136.3	17.7	*t*_*4*_=3.593	P_*4*_=0.000
	no myocardial injury group	78	87.2	19.1		
The sixth cycle of chemotherapy group	myocardial injury group	4	140.1	14.8	*t*_*5*_=5.620	P_*5*_=0.000
	no myocardial injury group	69	90.1	17.4		

The data from the second cycle of chemotherapy were excluded because only one patient developed into myocardial injury which is not suitable for t test.

**Figure 2:**
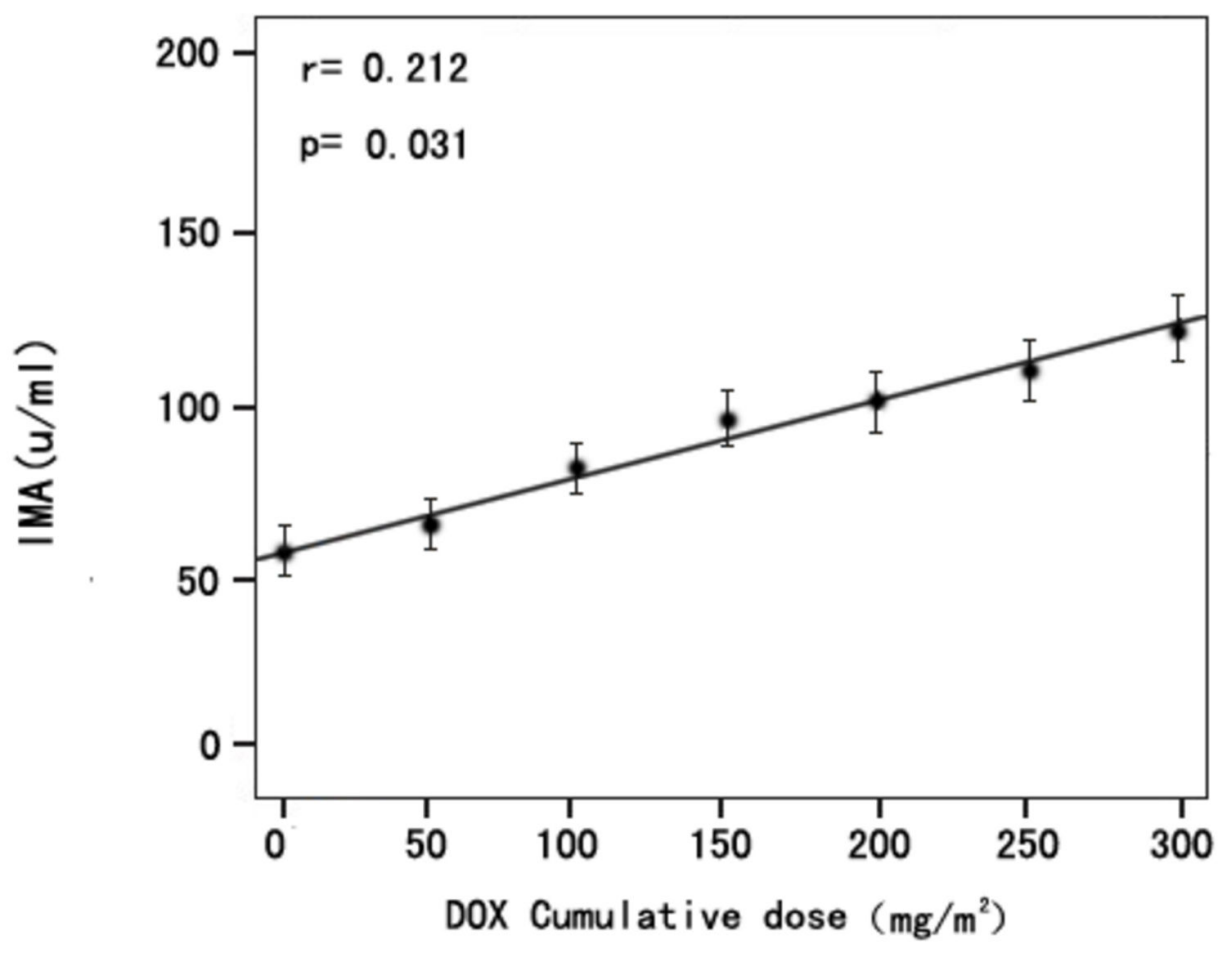
The linear correlation analysis of the cumulative dose of DOX and serum IMA measured at each cycle of chemotherapy. IMA positively correlated with the cumulative dose of DOX (*r* = 0.212, *P* < 0.05).

The cumulative dose of DOX in 1to 6-cylcle of chemotherapy is respectively 50mg/m^2^, 100mg/m^2^, 150mg/m^2^, 200mg/m^2^, 250mg/m^2^ and 300mg/m^2^. The mean IMA concentration of serum samples after each cycle of chemotherapy and the cumulative dose of DOX in 1to 6-cylcle of chemotherapy were analyzed by the linear correlation analysis. The results showed that the increased IMA concentration positively correlated with the cumulative dose of DOX (r = 0.212, P = < 0.05; Figure 2). 

**Figure 3:**
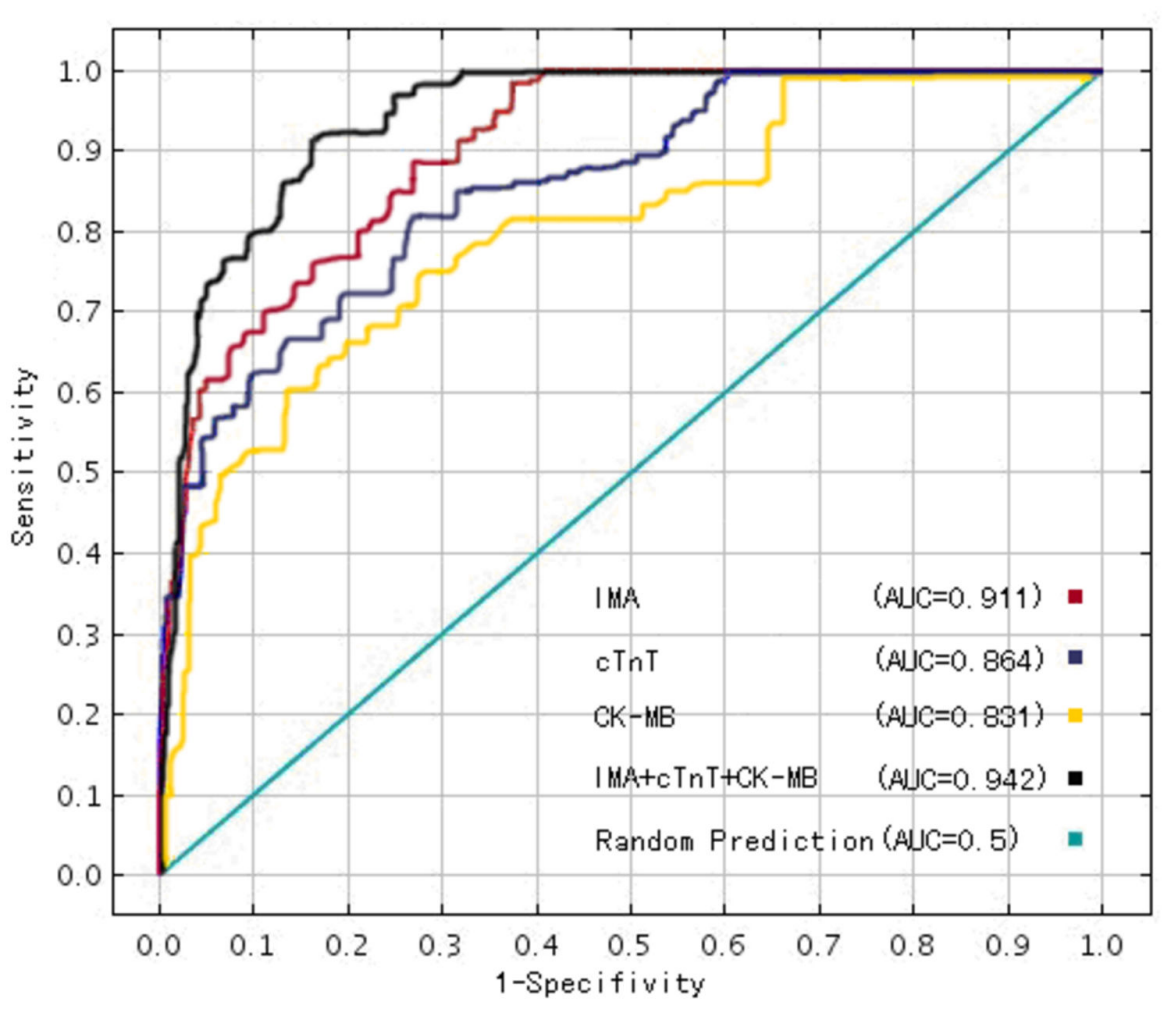
ROC curves analysis of IMA, cTnT, CK-MB, and IMA + cTnT + CK-MB. ROC analysis included 136 patients with no myocardial injury as a negative group and 16 patients with myocardial injury as a positive group. Receiver Operating Characteristic curves: ROC curves; AUC: area under the ROC curves.

The sensitivity and specificity of IMA were determined by ROC curves, and AUC were calculated [15]. The AUC for IMA was 0.911 (95% confidence interval [95% CI]; range, 0.876–0.946). The optimal cutoff concentration for IMA was 112.09 U/ml, which had a sensitivity of 0.891 and a specificity of 0.723. The AUC for cTnT was 0.864 (95% CI; range, 0.821–0.905) and the AUC for CK-MB was 0.831 (95% CI; range, 0.785–0.887). The AUC for IMA combined with cTnT and CK-MB was 0.942 (95% CI; range, 0.920–0.964) (*z=2.07, P<0.05*, versus AUC value of IMA by z test), with a sensitivity of 0.920 and a specificity of 0.830 (Table 2 and Figure 3). 

**Table 2:** ROC curves analysis of IMA, cTnT and CK-MB.

	AUC	SEz	95% CI	Sensitivity	Specificity
IMA	0.911	0.015	0.876–0.946	0.891	0.723
cTnT	0.864	0.021	0.821–0.905	0.831	0.727
CK-MB	0.831	0.028	0.785–0.887	0.742	0.721
IMA+cTnT+CK-MB	0.942^*^	0.011	0.920–0.964	0.920	0.830

AUC: area under the ROC curves; SEz: Standard error; CI: Confidence interval

* *z=2.07, P<0.05*, versus AUC value of IMA by z test

The serums of 59 patients followed-up for one year were divided into Group A (mean IMA concentration /patient> 112.09 U/ml) and Group B (mean IMA concentration /patient < 112.09 U/ml) by comparing the mean IMA concentration of all cycles of post-chemotherapy in each patient with the optimal cutoff of 112.09 U/ml. The LVEF data were not normally distributed, but no significant difference was found between the groups before chemotherapy (*u* = 0.54, *P* = 0.295). In Group A, LVEF declined significantly in the year after the last cycle of chemotherapy (*u* = 2.19, *P* = 0.014). At one year after chemotherapy, there was a significant difference in the LVEF between Group A and B (*u* = 5.39, *P* = < 0.001; Table 3). The rate of change for the mean IMA concentration and LVEF were calculated for 59 patients. The rate of change for IMA was calculated according to the following formula:

([mean IMA concentration]_pre-chemotherapy_ – [mean IMA concentration]_post-chemotherapy_) / [mean IMA concentration]_pre-chemotherapy_

**Table 3:** LVEF changes in Group A and B.

Group	n	Pre-chemotherapy LVEF median (%)	Post-chemotherapy at 12 month LVEF median (%)
A	12	67.1(55.3–70.1)	58.3(45.3–64.3)^*^
B	47	66.3(56.4–69.3)	63.5(48.1–68.2)^▲^

* *u* = 2.33, *P* = < 0.001, *versus* group A pre-chemotherapy by Wilcoxon singed rank test; ▲*u* = 5.39, *P* = < 0.001, *versus* group A post-chemotherapy by Wilcoxon rank sum test.

**Figure 4:**
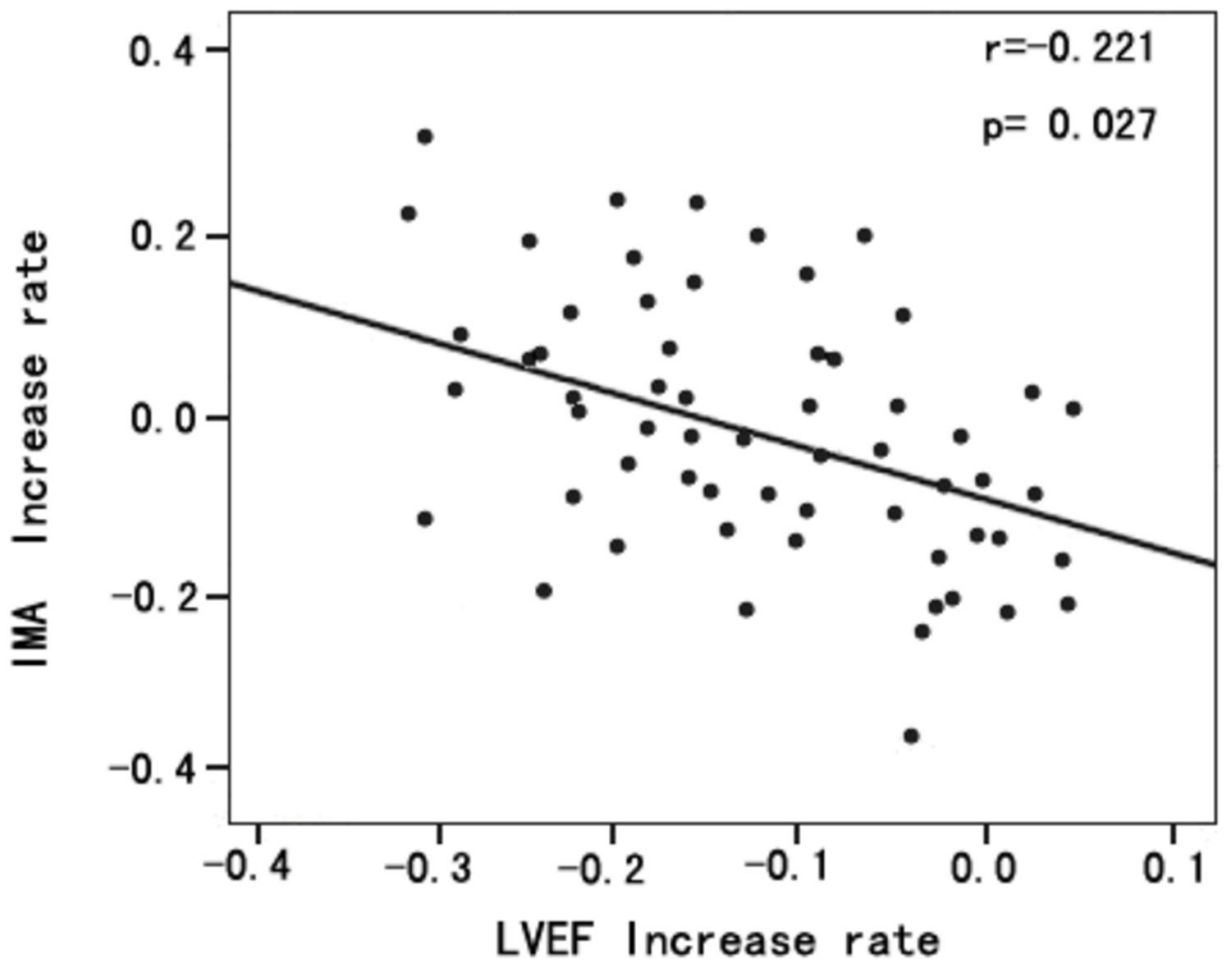
Spearman univariate correlation analysis between the rate of change for IMA with the rate of change for LVEF. The rate of change for IMA was negatively associated with the rate of change for LVEF (*r* = –0.221, *P* = 0.027).

The rate of change for LVEF before chemotherapy and at the one year follow-up was calculated in the same way. Correlation analysis revealed that the rate of change for IMA correlated negatively with the rate of change for LVEF (*r* = –0.221, *P* = 0.027; Figure 4).

## 4. Discussion

DOX-induced myocardial injury may be acute, sub-acute or chronic. Acute or sub-acute cardiac injury occurs during, or immediately after administration of doxorubicin; is caused by cardiac myocyte edema and vacuolar degeneration; and is seen on ECG as non-specific ST-T segment changes, low amplitude QRS complexes and a prolonged QT interval. Two types of chronic cardiac injury are recognized: early chronic progressive injury, also called tardive myocardial injury reaction, which occurs within a year of drug withdrawal; and late chronic progressive cardiac injury, which can occur one year, several years or decades later [16,17]. Pathologically, these cases are characterized by myofibril lysis, focal myocardial lysis, irreversible cardiomyocyte collagen deposition or interstitial fibrosis [18].It is irreversible when cardiac injury was diagnosed by ECG and pathology. This damage is further exacerbated by high doses of the drug [19,20]. Because of the potential for cardiac injury, the dose of DOX should be carefully controlled. If DOX is used by combining with another drug, long-term survival is best when the dose of DOX does not exceed 300 mg/m^2^[21] . However, along with improved chemotherapy success rates, life expectancy and quality of life in patients with breast cancer by controlling the dose of DOX or combining other drugs, the incidence of DOX-induced cardiac injury has also increased. ECG changes can be detected over time and the incidences of cardiomyopathy, in particular, and heart failure, in general, rise [22,23]. It is important to prognosticly evaluate DOX-induced myocardial injury before cardiac damage becomes extensive and irreversible as there is no specific treatment. 

IMA can be detected in the early stages of myocardial ischemia before the onset of cardiomyocyte necrosis. IMA is formed when serum albumin passes through ischemic tissues, causing structural changes at the N-terminal free metal binding domain that consists of an aspartic acid-alanine-histidine-lysine amino acid sequence. During ischemia and oxidative stress, oxygen free radicals damage two to four of these amino acids [9-11] and both the hydroxyl radical (•OH) and Fenton reaction play an important role in this process [8]. Numerous studies have confirmed that increases in the concentration of serum IMA are associated with myocardial ischemia and injury, and correlate with ECG changes [24,25].

No previous studies have examined the relationship between changes in serum IMA concentration and DOX-induced myocardial injury. So we are ready to measure serum IMA in patients with breast cancer before and after each cycle of DOX chemotherapy and calculated the cumulative dose of DOX. However, albumin cobalt binding assay determines IMA concentration based on the unbound cobalt to albumin, IMA concentration prediction can’t be accurate unless it is determined with respect to total albumin concentration in the patients before and after DOX treatment. So the 152 serum samples of 152 patients before chemotherapy and serum samples of patients after each cycle of chemotherapy were measured for serum albumin concentration, the serum albumin concentration of patients before DOX chemotherapy and the serum albumin concentration of patients after each cycle of DOX chemotherapy (serum albumin concentration both at 35.0-55.0 g/L) were analyzed before detecting the IMA levels. There was no significant difference for serum albumin concentration before and after each cycle of DOX chemotherapy (data not shown). Then excluding the serum albumin concentration factor, we detected the IMA levels. The results showed that the pre-chemotherapy serum concentration of IMA was (59.2 ± 10.9) U/ml, which was nearly consistent to the serum concentration of IMA for the control reference population in a previous study [26] (Figure 1 ). There was significant difference for serum IMA concentrations before and after each cycle of DOX chemotherapy. Then the serum samples of patients after each cycle of chemotherapy were divided into myocardial injury group and no myocardial injury group according to the diagnosis criteria of myocardial injury. We found that there was significant difference of IMA concentration in serum samples between myocardial injury group and no myocardial injury group in each cycle of chemotherapy (Table 1), And the serum IMA concentration positively correlated with the cumulative dose of DOX (Figure 2). 

In order to further evaluate the clinical value of IMA, we compared the serum IMA concentrations with cTnT and CK-MB. After 1-year follow-up, when serum IMA were combined with cTnT and CK-MB levels, the sensitivity and specificity(with a sensitivity of 0.920 and a specificity of 0.830 )for diagnosing DOX myocardial injury were improved, and the AUC (0.942 [95% CI; range, 0.920–0.964])was largest (Table 2 and Figure 3). After follow-up at 1 year, we found that the impairment in cardiac function was most pronounced in patients whose IMA increase exceeded the cutoff value (Table 3). Moreover, rate of change for IMA was negatively correlated with the rate of change for LVEF (Figure 4).

As an anthracycline antibiotic, DOX exhibits a high affinity for membrane phospholipids (leaflet lipid, liposome), neutral phospholipids (lecithin, egg lecithin), and cardiolipin, resulting in a very long elimination half-life from cardiac tissue [27]. Our observations also confirm a link between myocardial injury and raised serum IMA levels, suggesting the presence of increased oxidative stress in this group (myocardial injury group)[28,29]. Impaired cardiac function was also seen by ST-T, T and Q-T of changes on ECG in patients with the higher cumulative dose of DOX, and with higher serum IMA concentration. Our findings suggest that an increased serum IMA concentration can be helpful to predict DOX-induced myocardial injury. Numerous studies have demonstrated that the levels of oxygen free radicals were higher in patients with DOX-induced myocardial injury [28,29]. Oxidative stress is one of the main pathogenesis of heart failure, the production of ROS caused myocardial dysfunction in myocardial injury and necrosis. And IMA increasing is related to ROS in myocardial ischemia reperfusion process ,so IMA level may reflect severe acute myocardial dysfunction [30,31]. The levels of serum IMA correlate closely with the severity of myocardial ischemia and hypoxia which induced oxidative stress, but not age, gender, history of myocardial infarction, hypertension, hyperlipidemia, or the number or extent of diseased or stenotic coronary vessels [20,32]. Pregnancy, infection and an abnormal serum albumin concentration can affect IMA levels [33] and these potentially confounding factors were exclusion criteria in our study. IMA levels increase in athletes 24–48 hours after a marathon [34], presumably as a consequence of late gastrointestinal or skeletal muscle ischemia [35]. IMA levels also increase in patients with systemic cirrhosis and shock, which reflects global oxidative stress, and in patients with end-stage renal failure and tumors, but not in patients with traumatic injury, musculoskeletal or peripheral vascular disease [34–36]. However, the extent of the IMA concentration increase in these conditions is less than with myocardial damage [37–40].

In conclusion, we found that IMA is a valuable indicator of diagnosing DOX-induced myocardial injury and is helpful to predict long-term impairment of cardiac function for DOX-induced myocardial injury patients with breast cancer. However, whether IMA can really work as a marker of chronic toxicity (long term over years) from DOX in clinical, further studies are needed by broaden numbers of patients and a randomized control study. In addition, we studied serum IMA only in patients with breast cancer, and caution must be exercised when extrapolating our findings to patients receiving DOX for other indications. And our conclusion based on clinical observation rather than laboratory-based mechanism research, further studies also are needed to broaden the research scope to include other tumor types and to explore the mechanism of IMA generation in DOX-induced myocardial injury.
